# Integrative Analyses of Transcriptomes to Explore Common Molecular Effects of Antipsychotic Drugs

**DOI:** 10.3390/ijms23147508

**Published:** 2022-07-06

**Authors:** Trang T. T. Truong, Chiara C. Bortolasci, Srisaiyini Kidnapillai, Briana Spolding, Bruna Panizzutti, Zoe S. J. Liu, Jee Hyun Kim, Olivia M. Dean, Mark F. Richardson, Michael Berk, Ken Walder

**Affiliations:** 1 The Institute for Mental and Physical Health and Clinical Translation (IMPACT), School of Medicine, Deakin University, Geelong 3220, Australia; truongtra@deakin.edu.au (T.T.T.T.); chiara.bortolasci@barwonhealth.org.au (C.C.B.); srisaiyini.kidnapillai@med.lu.se (S.K.); briana.spolding@deakin.edu.au (B.S.); b.panizzuttiparry@deakin.edu.au (B.P.); zoe.liu@deakin.edu.au (Z.S.J.L.); jee.kim@deakin.edu.au (J.H.K.); o.dean@deakin.edu.au (O.M.D.); michael.berk@deakin.edu.au (M.B.); 2 The Florey Institute of Neuroscience and Mental Health, University of Melbourne, Parkville 3010, Australia; 3 Genomics Centre, School of Life and Environmental Sciences, Deakin University, Burwood 3125, Australia; m.richardson@deakin.edu.au; 4 Orygen, The National Centre of Excellence in Youth Mental Health, Centre for Youth Mental Health, The Florey Institute for Neuroscience and Mental Health and the Department of Psychiatry, University of Melbourne, Parkville 3010, Australia

**Keywords:** transcriptomics, gene expression, schizophrenia, antipsychotics, psychiatry, mental disorders

## Abstract

There is little understanding of the underlying molecular mechanism(s) involved in the clinical efficacy of antipsychotics for schizophrenia. This study integrated schizophrenia-associated transcriptional perturbations with antipsychotic-induced gene expression profiles to detect potentially relevant therapeutic targets shared by multiple antipsychotics. Human neuronal-like cells (NT2-N) were treated for 24 h with one of the following antipsychotic drugs: amisulpride, aripiprazole, clozapine, risperidone, or vehicle controls. Drug-induced gene expression patterns were compared to schizophrenia-associated transcriptional data in post-mortem brain tissues. Genes regulated by each of four antipsychotic drugs in the reverse direction to schizophrenia were identified as potential therapeutic-relevant genes. A total of 886 genes were reversely expressed between at least one drug treatment (versus vehicle) and schizophrenia (versus healthy control), in which 218 genes were commonly regulated by all four antipsychotic drugs. The most enriched biological pathways include Wnt signaling and action potential regulation. The protein-protein interaction (PPI) networks found two main clusters having schizophrenia expression quantitative trait loci (eQTL) genes such as *PDCD10*, *ANK2,* and *AKT3*, suggesting further investigation on these genes as potential novel treatment targets.

## 1. Introduction

Despite pharmacological advances, drug discovery for schizophrenia remains a formidable challenge due to the fact that the etiopathogenesis of the disease is poorly understood. To accelerate novel drug discovery, identifying potential molecular targets from the expanding genetic and transcriptomic data is a promising approach, especially given the rise of high throughput sequencing technologies and datasets. Schizophrenia is highly heritable, and several genome wide association studies (GWAS) have identified risk loci related to the disease [1,2,3]. Similar to schizophrenia pathogenesis, the molecular mechanisms of widely used antipsychotic drugs are likely polygenic, with probable overlap between schizophrenia risk loci and antipsychotic drug target genes [4]. Hence, mechanistic insights into how antipsychotic drugs ameliorate symptoms may be gained by investigating antipsychotic-induced alterations in gene expression, especially in schizophrenia risk genes. Importantly, drugs with a supported genetic mechanism tend to perform better in clinical development than those without such evidence, with an estimated doubling in success rate from phase I to approval [5,6].

While transcriptional profiling of psychotropic drug treatments offers a comprehensive view of cellular response at molecular level [7], identification of the relevant perturbations driving the beneficial therapeutic effects can be challenging. However, the integration of post-mortem transcriptional profiles with gene expression changes induced by widely prescribed antipsychotic drugs (i.e., amisulpride, aripiprazole, clozapine, risperidone) enable us to focus on latent potential molecular targets underlying the drugs’ therapeutic effects. Since these drugs are expected to alleviate at least some of the aberrant perturbations in schizophrenia, genes that are regulated by the drug in the opposite direction from the disease phenotype may be therapeutic targets. Identifying such genes with reversed regulation by the drug compared to the disease in their expression has been applied successfully for drug discovery in oncology, in which it was shown that the extent of reversed disease gene expression correlated with the drug efficacy [8]. While this approach is new in psychiatry, a study has shown overexpressed genes in schizophrenia patients reverting to normal expression levels after antipsychotic drug treatment [9], supporting the use of transcriptomic reversal to identify molecular targets underlying the efficacy of antipsychotic drugs. 

**Figure 1:**
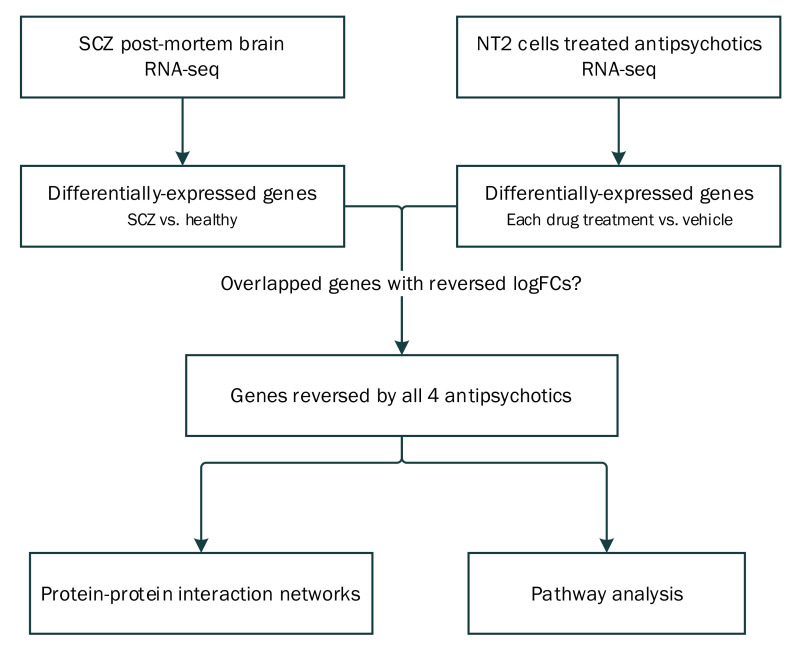
Flowchart of the analytical approach of the current study. Schizophrenia-associated transcriptional perturbations were compared against drug-induced gene expression profiles to identify potential therapeutic-relevant genes that were reverse regulated by all four antipsychotic drugs (i.e., amisulpride, aripiprazole, clozapine, risperidone). Pathway analysis on these genes then found enriched pathways potentially relevant to the molecular mechanism(s) of antipsychotics. Protein-protein interaction analysis was also applied for the commonly reversed genes by all antipsychotics to find their potential functional connections. Abbreviations: LogFC, log fold change relative to healthy control brains or vehicle treated cells; SCZ, schizophrenia.

In this study, antipsychotic-induced gene expression profiles were obtained using RNA-sequencing in an in vitro human neuronal cell model and examined with respect to schizophrenia-associated transcriptional data in post-mortem brain tissues. A summary of our investigative strategy is shown in Figure 1. The four widely-used atypical antipsychotics were selected from four different groups based on their receptor binding profiles: the serotonin and dopamine antagonists (risperidone), the multiple-acting receptor targeted antipsychotics (clozapine), the D_2_ partial agonists (aripiprazole), and others (amisulpride) [10,11]. Capitalizing on their different binding profiles, we aimed to evaluate their potential common effects at the transcriptional level and how they might interact in the protein-protein interaction networks, as well as their relevant biological processes. 

## 2. Results

### 2.1. Most Reversed Gene Expressions Were Changed by All Four Antipsychotic Drugs Rather than by a Single Antipsychotic Drug

**Figure 2:**
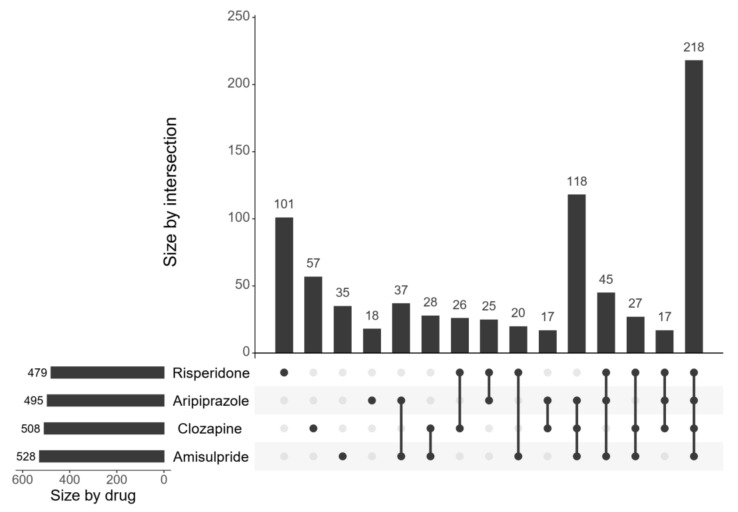
The number of genes whose expression levels were reversed by the individual and combination of antipsychotic drugs relative to the differential expression in post-mortem brains. The total number of genes with reverse regulated expression by each drug is shown on the leftmost horizontal axis. The vertical axis from the top panel demonstrates the unique sets of genes reversed by individual drugs or the intersection of these sets, indicated by the connected lines and dots along the horizontal axis.

In comparisons between transcriptional profiles of antipsychotics and schizophrenia post-mortem brains, 886 genes were found to be expressed in the opposite direction between at least one drug treatment (as compared to vehicle) and schizophrenia (as compared to healthy control). Figure 2 shows the number of genes reverse regulated by each drug and their intersections (i.e., shared by two or more drugs). Amisulpride reverse regulated the greatest number of genes (*n* = 528), while risperidone reverse regulated the least number of genes (*n* = 479). Interestingly, most gene expression reversals were driven by all four antipsychotic drugs (*n* = 218) rather than by any one antipsychotic.

### 2.2. Reverse Regulation of Genes Was Enriched for Pathways Related to Action Potential, Signal Transduction and Response to Insulin

**Figure 3:**
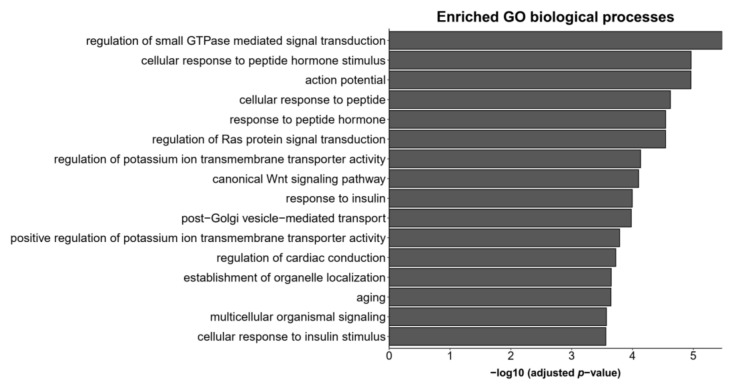
Gene ontology biological processes enriched by the list of genes reversed by all antipsychotic drugs.

The biological functions of 218 reverse regulated genes by all four drugs were evaluated based on functional enrichment analysis of gene sets from Gene Ontology (GO). GO enrichment analysis found 16 significantly enriched biological processes with *p*-values adjusted for multiple testing (FDR) < 0.05 (illustrated in Figure 3, full results provided in Supplementary Table S1). The results highlighted the involvement of action potential (e.g., “action potential”, “regulation of potassium ion transmembrane transporter activity”, “positive regulation of potassium ion transmembrane transporter activity”), signal transduction pathways (e.g., “regulation of small GTPase mediated signal transduction”, “regulation of Ras protein signal transduction”, “canonical Wnt signaling pathway”) and response to insulin (e.g., “cellular response to peptide hormone stimulus”, “cellular response to peptide”, “response to insulin”).

### 2.3. Gene Networks Identified Two Main Clusters Related to Wnt Signalling and Action Potential

**Figure 4:**
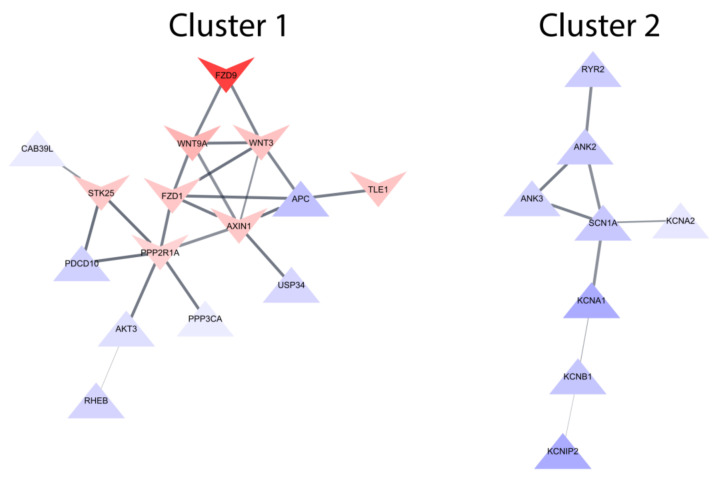
Clusters of protein-protein interaction networks of the commonly reversed genes by four antipsychotic drugs. Cluster 1 and 2 are the two largest connected components of biologically related proteins. Triangular nodes are genes upregulated by all studied antipsychotics, whilst v-shaped nodes represent downregulated genes. Nodes are colored based on mean log fold change (red: downregulation, blue: upregulation). Edge width represents the confidence of the STRING interaction score graded by supporting evidence (from cut-off minimum score 0.7 to maximum score 1). Proteins without interactions are not shown.

While pathway analysis offered useful insights on the enriched biological functions, it lacks the consideration of interactions between genes. We used the STRING database of protein-protein interactions (PPI) to find the potential intergenic interactions between protein-coding genes, filtering for only interactions with high confidence. The PPI network had a significant enrichment *p*-value of 0.00027, suggesting that commonly reversed genes have meaningful connections and are not a random set of genes. As shown in Figure 4, two major clusters were identified (fully constructed networks can be found in Supplementary Figure S1). Cluster 1 comprised of proteins related to signaling pathways: Wnt signaling pathway (TLE1, APC, WNT3, FZD1, FZD9, WNT9A, AXIN1, USP34, PPP3CA), Akt signaling pathway (AKT3, RHEB, PPP3CA) and the striatin-interacting phosphatase and kinase (STRIPAK) signaling complex (PDCD10, PPP2R1A, STK25). Cluster 2 contained solely upregulated proteins involved with action potential regulation: potassium channel (KCNIP2, KCNB1, KCNA1, KCNA2), sodium channel (SCNA1), calcium channel (RYR2) and ion channel stabilization (ANK2, ANK3). 

Expression quantitative trait loci (eQTL) are single nucleotide polymorphisms (SNPs) that account for some of the variance in RNA expression across conditions, and integration of eQTLs with genome-wide association studies (GWAS) data has identified risk genes for schizophrenia [12]. To complement the robustness of finding therapeutic relevant gene targets, we compared with evidence from GWAS and found three schizophrenia-associated eQTL genes in the two main clusters of the PPI networks (i.e., *PDCD10* [13] and *AKT3* [14] in cluster 1, *ANK2* [14] in cluster 2).

## 3. Discussion

In this study, we highlighted the common targets and pathways regulated by four commonly prescribed antipsychotic drugs. We used the integration of our RNA-sequencing results from in vitro treatments with expression profiling datasets of post-mortem brain samples from people with schizophrenia. Despite the unique transcriptional features of each antipsychotic drug, our study pinpointed the common reversal effects versus schizophrenia phenotypes of 218 genes by all four antipsychotic drugs. Through our multi-stepped bioinformatics analyses, signaling pathways and action potential regulation were highlighted as the major biological processes underlying the common mechanisms of the studied antipsychotic drugs. 

STRING protein-protein interaction (PPI) network of proteins encoded by genes reversely regulated by all four antipsychotic drugs implied association with signaling pathways (Figure 4—Cluster 1), including elements of the Wnt pathway and the Akt pathway. Abnormal Wnt pathway signaling in schizophrenia is documented, together with its association with the Akt pathway [15,16,17]. One major element of the Akt pathway found in the network is *AKT3* (AKT Serine/Threonine Kinase 3), which is also an eQTL gene implicated in schizophrenia via GWAS [1,18]. *AKT3* was upregulated by all four antipsychotics, potentially reversing the downregulation seen in post-mortem brains from people with schizophrenia. *AKT3* is a member of the *AKT* family that play roles in many biological processes such as cell proliferation and apoptosis [19]. *AKT3* is the most abundant *AKT* isoform in the brain during neurogenesis [20,21]. It has a major role in brain development and the AKT3 knock-out mice exhibited a phenotype reminiscent of psychiatric manifestations including schizophrenia [22,23,24].

In cluster 1 of the PPI network, the STRIPAK signaling complex could be highly relevant to the common mechanisms of the studied antipsychotics, given its association with elements of the Wnt pathway and the Akt pathway. We found three genes of STRIPAK which were reversely regulated by all four antipsychotic drugs. These were the scaffolding subunit (*PPP2R1A*), germinal center kinase GCKIII (*STK25*) and GCKIII tether (*PDCD10*). While STRIPAK has not been researched in schizophrenia, a variety of proteins can be phosphorylated by STRIPAK complexes to regulate multiple cellular processes including signaling pathways such as Hippo signaling [25,26], whose modulation by antipsychotic drugs has been previously described [27]. Among these STRIPAK genes of the PPI network, *PDCD10* (Programmed Cell Death 10) is an eQTL gene associated with schizophrenia risk [13]. This was upregulated by all four antipsychotics, reversing the downregulation seen in post-mortem brains from people with schizophrenia. In JM-Jurkat T-lymphocytes, *PDCD10* was up-regulated by both acute and subacute treatment of clozapine in a microarray experiment as well as qPCR [28]. Evidence so far suggests *PDCD10* has pleiotropic involvement in apoptosis, oxidative metabolism, Golgi complex polarization, and especially in cerebral cavernous malformation disease [29,30]. However, it remains unknown how this gene might contribute to the pathology of schizophrenia.

Action potential regulation was another major common mechanism of antipsychotic drugs in this study. The PPI network identified a cluster of upregulated genes involved in action potential (Figure 4—Cluster 2), in which most are elements of potassium channels, concurring with its major significance in GO enrichment analysis enriching relevant processes such as “action potential” and “regulation of potassium ion transmembrane transporter activity”. Ion channel stabilizers (i.e., *ANK2* and *ANK3*), might play an interconnected role affecting multiple ion channels in the cluster. Indeed, *ANK3* (Ankyrin 3) binds to sodium channels and potassium channels, and is required for the organization of these elements at the axon initial segment for normal action potential firing [31,32]. While the function of *ANK2* in brain has not been as well researched as *ANK3*, *ANK2* is a schizophrenia-related eQTL gene [14]. Its antipsychotics-induced upregulated expression was reversed compared to the downregulation trend observed in post-mortem brains from people with schizophrenia. *ANK2* tends to be co-expressed with *ANK3* in many cell types including neurons, and both *ANK2* and *ANK3* might play important roles in axonal trafficking [33]. However, the biological functions in schizophrenia of both ankyrins remain to be fully characterized. 

While the evaluation of antipsychotic drug effects on gene expression have been reported in some studies [34,35], these results were acquired from limited drug treatments and gene coverage. The strength of this study was the usage of RNA sequencing for multiple drugs, enabling us to compare different drugs on a more comparable level due to our same experimental protocol and obtain a higher level of coverage of gene expression levels from high-throughput sequencing. The integrative analyses with transcriptional profiles from schizophrenia individuals also add clinical corroboration to the identification of molecular targets underlying the efficacy of antipsychotic drugs via transcriptomic reversal. Whilst the evaluation of the exclusive transcriptional features of each antipsychotic drug may yield interesting results, this study did not aim to examine these specific expression changes and hence they should be considered separately. We acknowledge some limitations of this study. The NT2-N in vitro model is an imperfect representation the disease phenotype. In our NGS analyses, we administered a single dose for each drug with acute measurement, which limits our ability to assess long-term pharmacological regulation. There is a possibility that the post-mortem brain dataset used in this study was influenced by drug treatments. However, Fromer et al. has examined enrichment of differential expression and directional concordance for drug treatment signatures derived from studies using monkeys and rodents and found genes highlighted by the contrast of subjects with schizophrenia versus control subjects do not largely trace their differential expression to antipsychotic medications [36]. The most ideal study design would be pre- and post-treatment measurement in drug naïve schizophrenia patients versus healthy controls. To our knowledge, one study by Benedicto et al. has characterized differentially expressed genes in blood between schizophrenia patients before and after treatment with atypical antipsychotics using next-generation sequencing data [9]. Despite its accessibility, human whole blood is a limited substitute for brain tissue, affecting the characterization of transcriptional dysregulation in mental disorders [37,38]. Nevertheless, post-mortem brain tissues from schizophrenia subjects still offer most acceptable reflection of gene expression associated with the disorder [39]. 

## 4. Materials and Methods

### 4.1. Post-Mortem Brain Gene Expression Datasets

The gene expression dataset from Fromer et al. [36] was utilized, in which dorsolateral prefrontal cortex post-mortem brain tissues of people diagnosed with schizophrenia (*N* = 258) and controls (*N* = 279) were sequenced by RNA-seq. A total of 56,632 genes were sequenced and among them, there were 16,423 genes passing the expression-level threshold of >1 CPM in >50% of the samples. Applying a significant cut-off FDR of 0.1, we extracted 794 genes that were differentially expressed with their corresponding logFCs. 

### 4.2. Cell Culture

The model of human neurons used in our study was NTera2/cloneD1 (NT2)—a human teratocarcinoma cell line, which was differentiated into post-mitotic neuronal cells (NT2-N) after being treated with retinoic acid [40,41]. The NT2-N cells share many characteristics of human embryonic stem cells and of neuronal progenitor cells [42] and have been an efficient proxy for the study of central nervous system biology in various disorders such as Lesch-Nyhan disease and Parkinson’s disease [43,44]. The cell culture and differentiation processes were conducted as previously described [45]. Briefly, NT2 cells were maintained in Dulbecco’s modified Eagle’s Medium (DMEM; Life Technologies, Melbourne, Australia), 10% fetal bovine serum (FBS; Thermo Fisher Scientific, Melbourne, Australia) and 1% antibiotic-antimycotic solution (Life Technologies, Thermo Fisher Scientific, Melbourne, Australia). NT2-N cells were induced from NT2 cell cultures by treating with 10^−5^ M retinoic acid (Sigma-Aldrich, Sydney, Australia) for 28 days with media refreshed every 2–3 days. For experiments, cells were seeded onto plates coated with 10 μg/mL poly-D-lysine (Sigma-Aldrich, Sydney, Australia) and 10 μg/mL laminin (Sigma-Aldrich, Sydney, Australia) at 2 × 10^5^ cells/well (24-well plates) with further addition of mitotic inhibitors (1 µM cytosine and 10 µM uridine; Sigma-Aldrich, Sydney, Australia) for a total of 7 days, and the media was refreshed every 2–3 days to generate an enriched culture of differentiated neuronal cells (NT2-N). 

### 4.3. Drug Treatments

The differentiated neuronal cells were treated with one of the antipsychotic drugs: amisulpride (10 µM), aripiprazole (0.1 µM), clozapine (10 µM), risperidone (0.1 µM) for 24 h (4–6 replicates for each group). All drugs were purchased from Sigma-Aldrich (Sydney, Australia). Vehicle control cells were treated with an equal volume of 0.1% dimethyl sulfoxide (DMSO; Sigma-Aldrich, Sydney, Australia). These drug doses were chosen to maintain the exposure level within the therapeutic index according to previous dose response studies in our laboratory (data not shown).

### 4.4. Gene Expression Measurement

After the 24-h drug treatment, cells were harvested for RNA analysis. Total RNA was extracted using RNeasy^®^ mini kits (Qiagen, Melbourne, Australia), then checked for quality and quantity using an Agilent 2100 Bioanalyzer (Agilent Technologies, Melbourne, Australia) and NanoDrop 1000 (Thermo Fisher Scientific, Melbourne, Australia) respectively. 

RNA-seq libraries were prepared for all samples from 1 µg total RNA using a TruSeq RNA Sample Preparation Kit (Illumina, Victoria, Australia) as per the manufacturer’s instructions. Samples were analyzed on an Illumina HiSeq platform (HiSeq 2500 rapid 50 bpSE; 1 flow cell, 2 lanes) to measure genome wide mRNA expression.

### 4.5. Gene Expression Analysis

The raw data yielded in FASTQ format were aligned to reference genomes using the Deakin Genomics Centre RNA-Seq alignment and expression quantification pipeline (https://github.com/m-richardson/RNASeq_pipe, accessed on 1 July 2017). Briefly, the pipeline included raw read quality filtering and adapter trimming (ILLUMINACLIP:2:30:10:4, SLIDINGWINDOW:5:20, AVGQUAL:20 MINLEN:36) with Trimmomatic v35 (Usadel Lab, Jülich, Germany) [46], and alignment to the reference genome using STAR v2.5 (Cold Spring Harbor Laboratory, New York, USA) in 2-pass mode (Human genome version GRCh38) [47]. 

The expression was quantified at the gene level, and individual sample counts were collated into a m × n matrix for differential abundance testing. Low expressed genes were removed (<1 cpm in *n* samples, where *n* is the number of samples in the smallest group for comparison), and the data was normalized using the weighted trimmed mean of M-values (TMM) using edgeR in R [48]. Differential gene expression was assessed using edgeR, and statistical significance was corrected for multiple testing to generate false-discovery-rate (FDR) adjusted q-values via the Benjamini-Hochberg method [49].

### 4.6. Functional Enrichment Analysis and Protein-Protein Interaction Analysis

Using the differentially expressed genes identified from drug exposures and schizophrenia post-mortem brains, we identified overlapped genes with opposite directions of expression regulation (i.e., log fold change—logFC) between disease phenotypes and drug exposures (e.g., differentially expressed genes up-regulated in schizophrenia post-mortem brains but down-regulated by drug treatment in NT2-N cells). In order to find enriched pathways that might play a role in the molecular mechanism of antipsychotic drugs, we then filtered genes that were reverse regulated by all antipsychotic drugs, and applied over-representation analysis using the R package ClusterProfiler [50] with pathway reference from the gene ontology (GO) database [51].

The list of the commonly reversed genes induced by all antipsychotic drugs was also evaluated for protein–protein interactions (PPI) using the STRING database [52]. The interaction score minimum cut-off was set at 0.7 to filter only high-confidence connections.

## 5. Conclusions

In our study, we identified the main biological pathways involved in the beneficial effects of antipsychotic drugs and common linked genes. The highlighted pathways include Wnt signaling and action potential regulation. Some schizophrenia eQTL genes such as *PDCD10*, *ANK2* and *AKT3* with highly connected functions to these pathways should be further investigated to find out how they and their related pathways are involved with the disease, which might potentially enable the finding of novel treatment targets.

## Data Availability

The data that supports the findings of this study are available in the Supplementary Materials of this article and from the corresponding author upon reasonable request.

## Supplementary Materials

The following supporting information can be downloaded at: https://www.mdpi.com/article/10.3390/ijms23147508/s1.
