# Supplementary material for: Integrative Analyses of Transcriptomes to Explore Common Molecular Effects of Antipsychotic Drugs
Source: Int J Mol Sci. 2022 Jul 6;23(14):7508. doi: 10.3390/ijms23147508 (PMC9325239; doi:10.3390/ijms23147508)
Supplement: Supplementary file 1 [file ijms-23-07508-s001.zip › Supplementary Figure S1.pdf]

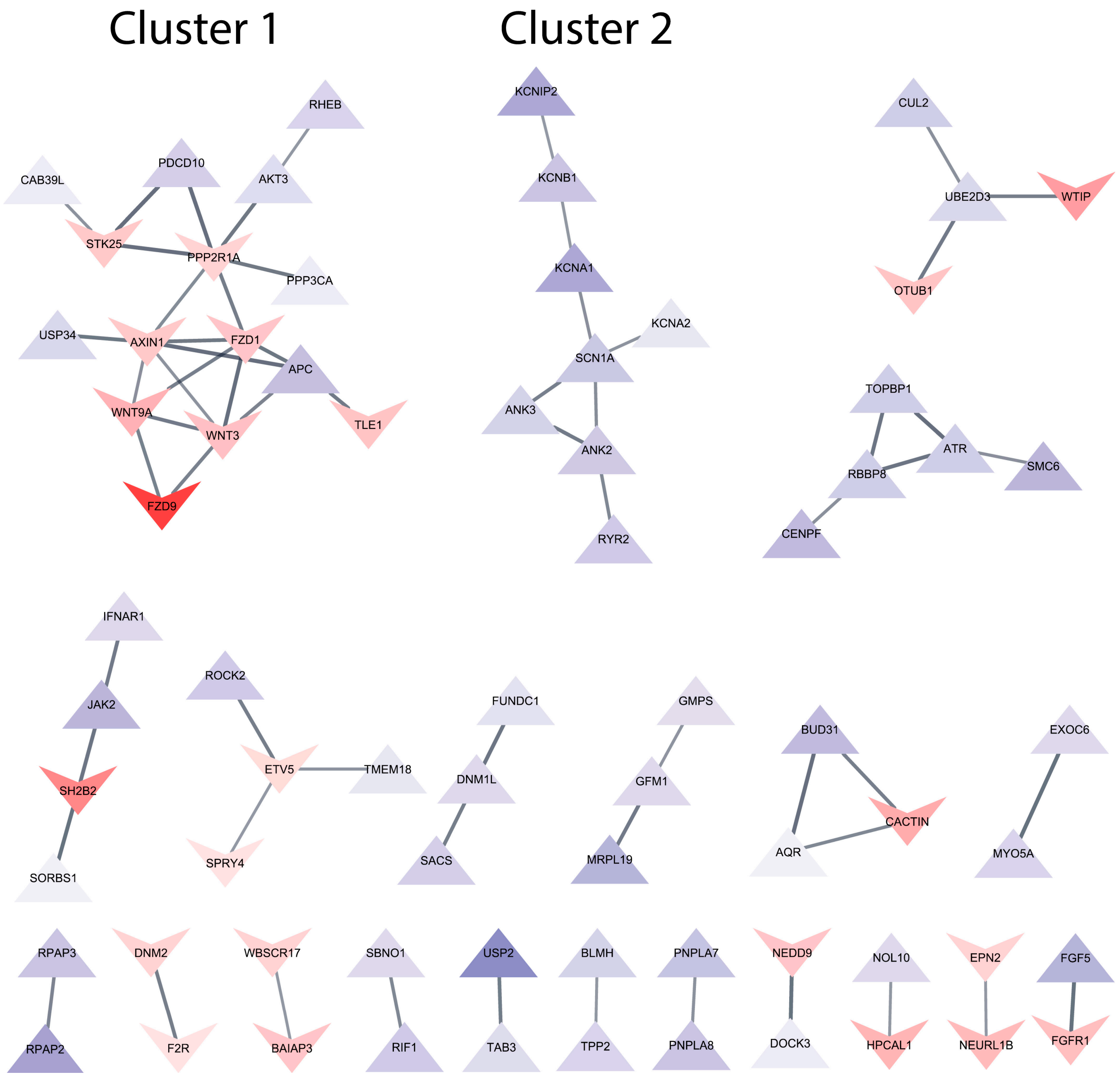

Figure S1. Fully constructed protein-protein interaction networks of the commonly reversed genes by four antipsychotic drugs.
